# A survey of offloading practices for diabetes-related plantar neuropathic foot ulcers

**DOI:** 10.1186/s13047-014-0035-8

**Published:** 2014-08-13

**Authors:** Anita Raspovic, Karl B Landorf

**Affiliations:** 1 Department of Podiatry, La Trobe University, Bundoora, Melbourne 3086, Australia; 2 Lower Extremity and Gait Studies Program, La Trobe University, Bundoora, Melbourne 3086, Australia

**Keywords:** Diabetic foot, Foot ulcer, Diabetic neuropathies

## Abstract

**Background:**

Offloading is key to preventing or healing plantar neuropathic foot ulcers in diabetes. Total contact casts or walkers rendered irremovable are recommended in guidelines as first-line options for offloading, however the use of such devices has been found to be low. This study aimed to investigate offloading practices for diabetes-related plantar neuropathic ulcers.

**Methods:**

An online survey of closed and open-ended questions was administered via SurveyMonkey®. Forty-one podiatrists experienced in high-risk foot practice, from 21 high-risk foot services around Australia, were approached to participate.

**Results:**

The response rate was 88%. Participants reported using 21 modalities or combinations of modalities, for offloading this ulcer type. The most frequently used modalities under the forefoot and hallux were felt padding, followed by removable casts or walkers, then non-removable casts or walkers. Participants indicated that many factors were considered when selecting offloading modality, including: compliance, risk of adverse effects, psycho-social factors, restrictions on activities of daily living, work needs and features of the wound. The majority of participants (83%) considered non-removable casts or walkers to be the gold-standard for offloading this ulcer type, however they reported numerous, particularly patient-related, barriers to their use.

**Conclusions:**

Selecting offloading for the management of foot ulceration is complex. Felt padding, not the gold-standard non-removable cast or walker, was reported as the most commonly selected modality for offloading plantar neuropathic ulceration. However, further evaluation of felt padding in high quality clinical trials is required to ascertain its effectiveness for ulcer healing.

## Background

Mechanical trauma is a key factor in the cause of many diabetes-related foot ulcers [1],[2]. Elevated peak plantar pressure, commonly measured in an attempt to quantify focal areas of mechanical trauma, is a significant risk factor for ulceration [3]-[5]. In turn, redistribution of plantar pressure away from sites that are elevated, commonly referred to as ‘offloading’, has been shown to result in faster healing of non-complicated neuropathic foot ulcers [1]. Accordingly, offloading is recommended as a primary therapy for the prevention and management of this ulcer type [6],[7].

Clinical guidelines recommend total contact casts (TCCs) or other devices rendered irremovable, such as below-knee walkers, as first line offloading options for the treatment of uncomplicated, diabetes-related neuropathic foot ulcers. There is evidence that such devices heal a significantly higher proportion of ulcers and also lead to faster healing [6],[7], but the use of these devices is not high, suggesting that there are important barriers to their use in everyday practice [8]-[10]. Where these interventions are not available, or if they are deemed inappropriate, ‘other’ offloading modalities should be considered, including: post-operative shoes, felt padding, half-shoes and cast-shoes [11]-[13]. However, the evidence for the effectiveness of ‘other’ offloading modalities for ulcer healing is limited [12].

Using evidence to select suitable offloading is further impeded by the narrow set of outcome measures, such as plantar pressure reduction and wound healing, that studies and subsequent guidelines have focused on. Potentially important psychosocial influences have not received adequate attention, but arguably have a strong impact on offloading suitability. These include: the capacity to work, drive a car, perform housework, sleep comfortably, as well as issues relating to self-consciousness and finances. A recent high quality systematic review supports this issue acknowledging the need for increased inclusion of health-related quality of life measures in offloading research [14].

This study, therefore, was designed to explore these issues in the context of Australian high-risk foot services. The broad aim of this project was to investigate offloading practices for diabetes-related plantar neuropathic ulcers. The specific aims of the project were to investigate: the types of offloading modalities used, how frequently they were used, the influences for their selection, and the barriers that prevent the use of non-removable casts or walkers.

## Methods

### Study design

This study used a survey to document the types of offloading modalities that were used and to explore clinicians’ views on the topic.

### Participants

Approval for this research was granted from the La Trobe University Faculty of Health Sciences Human Ethics Committee (FHEC12/107). Participants were advised that by opening the electronic survey and completing it, they were providing their consent to be involved in the project. Forty-one podiatrists experienced in high-risk foot practice, from 21 high-risk foot services around Australia, were approached to participate. Each state of Australia and the Australian Capital Territory was represented in approximate proportion to respective population estimates. The response rate was 88% (i.e. 36 of 41 invited clinicians participated in the study). Table 1 shows the number of clinicians invited per state and the respective response rates.

**Table 1:** Survey participation rates according to state/territory population estimates

**State**	**Population estimate (million)**	**Number of podiatrists invited to participate**	**Number of podiatrists completing survey**
New South Wales	7.2	10	8
Victoria	5.5	10	10
Queensland	4.5	6	5
Western Australia	2.1	5	4
South Australia	1.3	4	4
Tasmania	0.5	4	4
A.C.T	0.3	2	1
Total	21.4	41	36

Note: (i) the proportion of participants in Queensland is slightly lower compared to other states due to a relatively lower presence of metropolitan, public sector, high-risk foot services, (ii) ACT = Australian Capital Territory.

As there is no standard accepted definition for a high-risk foot service in Australia, the following inclusion criteria were applied. A high-risk service was considered to: have direct access to a broad range of disciplines (e.g. endocrinology, pathology and medical imaging), routinely manage complex and chronic high-risk foot complications in particular foot ulceration, and be based in the public sector or public hospital system. In this study, clinicians working in high-risk services in main metropolitan areas only were included except for Tasmania where clinicians in large regional services were also included (due to the dispersion of podiatrists in that state).

Twenty-one experienced clinicians working in eligible high-risk services were originally identified by the researchers through professional networks. Senior clinicians were targeted initially. An initial invitation to participate was sent to the 21 clinicians via e-mail. Upon their approval, a participant information statement, consent form and link to the survey were then forwarded electronically. All 21 clinicians initially contacted were asked to refer a colleague from their service for participation in the study where possible, which increased final participant numbers to 41 in total. A minimum of 12 months experience managing high-risk foot patients, at a minimum of 2 days a week, was required for inclusion into the study. The same process of electronic communication was then followed for newly recruited participants.

### Survey

An online survey consisting of closed and open-ended questions was administered via SurveyMonkey®. An initial version of the survey was piloted with 5 podiatrists working in high-risk foot practice around Australia to gauge the relevance and clarity of the questions and the ease of use of the tool. Their feedback was used to make subsequent changes and the final survey consisted of a series of 12 open and close-ended questions in total. All questions on the survey related to non-infected, non-ischaemic, plantar neuropathic foot ulcers, which were located either under the plantar forefoot (i.e. under the metatarsophalangeal joints) or under the hallux. The survey focused on conservative mechanical offloading modalities and clinicians were asked to complete the questions as they related to their main place of high-risk foot practice (i.e. the practice that most requires use of offloading modalities for ulcer treatment).

A copy of the full survey is included in Additional file 1 with a brief overview provided below. Questions 1 and 2 of the survey asked clinicians to nominate types of offloading modalities used and their respective frequency of use (i.e. % of patients used on) for this ulcer type. Twelve types of offloading were pre-listed on the survey, with an option for clinicians to add other modalities not on the list or to report combinations of modalities used. Question 3 asked clinicians to reflect on common ways that their offloading choices might change as an ulcer ran its clinical course. Questions 4 to 7 focused on perceived factors affecting selection of offloading modalities. Questions 8 to 10 asked about non-removable offloading in the form of total contact casts (TCCs) or non-removable walkers. Clinicians were asked to indicate: if they considered these modalities to be the gold standard for offloading non-complicated plantar neuropathic ulcers (Question 8), the types of non-removable casts or walkers used (Question 9), and what barriers they believed existed to using non-removable offloading (Question 10). Finally, Question 11 asked participants to estimate the percentage of time they used their preferred offloading selection.

### Data and content analysis

Survey data were collected in SurveyMonkey® and transferred to the Statistical Package for the Social Sciences (SPSS) Version 21.0 (SPSS Inc., Chicago, IL) for analysis. Descriptive data were generated for closed-ended questions, including frequencies (Questions 1, 2, 8 and 11) and median and range (Questions 4 to 7). No adjustments or imputations were made where there were missing data and to reflect this, the response rate per question is noted in the results. Responses to the open-ended questions (Questions 3 and 10) were explored using conventional content analysis where categories and names of categories were generated directly from the data in order to describe the phenomenon in question [15].

## Results

### Types and frequency of offloading modalities used for non-complicated plantar neuropathic ulceration

Results for Questions 1 and 2 indicate that a wide variety of offloading modalities were selected by participants for the management of both plantar forefoot and plantar hallux ulcers, with 21 offloading options being reported overall (12 pre-specified modalities and 9 additional modalities nominated by participants). The proportion of participants that indicated using the respective modalities varied, ranging from 94% of clinicians using felt padding to 3% of clinicians using standard footwear with a prefabricated insole or a non-removable cast with a wheelchair.

**Figure 1:**
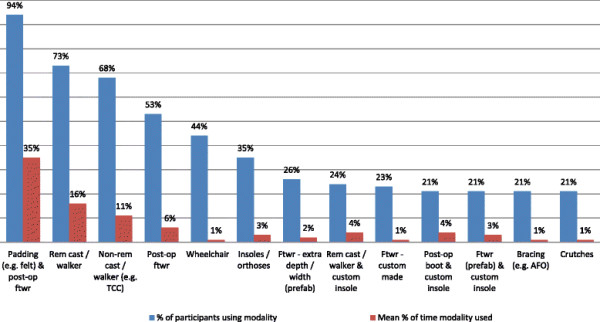
Offloading modality use for plantar forefoot ulcers (Question 1).

**Figure 2:**
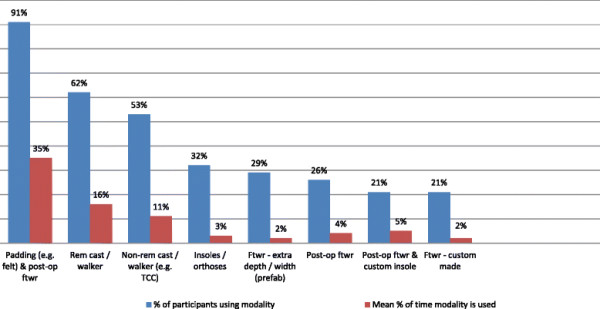
Offloading modality use for plantar hallux ulcers (Question 2).

The average percentage of time that each offloading modality was used also varied substantially between modalities (Tables 2 and 3, Figures 1 and 2). Padding (e.g. felt) with a post-operative shoe or footwear, a removable cast or walker, or a non-removable cast or walker were collectively used on average 62% of the time. For plantar forefoot and plantar hallux ulcers respectively, this equated to (in order of use): padding (felt/foam) being used by 94% and 91% of participants 35% of the time, removable casts or walkers being used by 73% and 62% of participants 16% of the time, and non-removable casts or walkers being used by 68% and 53% of participants 11% of the time. The other 18 offloading options were used for the remaining 38% of the time, but only by low percentages, ranging from 3 to 6%.

**Table 2:** Offloading modality use for plantar forefoot ulcers (Question 1)

**Offloading modality**	**% of participants using modality (n=)**	**Mean (±SD) % of time modality is used**
Padding (e.g. felt) with post-operative shoe or footwear	94 (32)	34.8 (22.5)
Removable cast/walker	73 (25)	16.1 (20.1)
Non-removable cast/walker (e.g. TCC)	68 (23)	11.2 (18.0)
Post-operative shoe	53 (18)	5.9 (8.2)
Wheelchair	44 (15)	1.1 (1.6)
Insoles/orthoses	35 (12)	2.7 (4.8)
Footwear - extra width/depth (prefabricated)	26 (9)	1.7 (3.2)
Removable cast/walker with custom insole	24 (8)	3.5 (3.2)
Footwear - fully custom made	23 (8)	1.4 (2.8)
Post-operative boot with custom insole	21 (7)	4.2 (2.9)
Footwear (prefabricated) with custom insole	21 (7)	2.5 (1.8)
Bracing (e.g. AFO)	21 (7)	0.7 (1.5)
Crutches	21 (7)	0.6 (1.4)

Note: modalities are ordered according to proportion of practitioners selecting the modality; N = 34; modalities used by less than 20% of participants (n = 7) are not listed.

**Table 3:** Offloading modality use for plantar hallux ulcers (Question 2)

**Offloading modality**	**% of participants using modality (n=)**	**Mean (±SD) % of time modality is used**
Padding (e.g. felt) with post-operative shoe or footwear	91 (31)	34.8 (23.6)
Removable cast/walker	62 (21)	16.0 (21.9)
Non-removable cast/walker (e.g. TCC)	53 (18)	10.8 (17.1)
Insoles/orthoses	32 (11)	3.4 (7.1)
Footwear - extra width/depth (prefabricated)	29 (10)	2.1 (3.9)
Post-operative shoe	26 (9)	3.8 (7.5)
Post-operative boot with custom insole	21 (7)	4.8 (3.5)
Footwear - fully custom made	21 (7)	1.5 (3.1)

Note: modalities are ordered according to proportion of practitioners selecting the modality; N = 34; modalities used by less than 20% of participants (n = 7) are not listed.

Of note was that the type of offloading modality selected and the percentage of time it was used did not differ substantially according to the location of the ulcer site. However, the proportion of participants selecting several offloading modalities reduced for hallux ulcers compared with forefoot ulcers. There was also a tendency towards greater frequency of use of insoles/orthoses, extra depth/width prefabricated footwear, post-operative boots with custom insoles, and prefabricated footwear with custom insoles for hallux ulcers.

### Influences on selection of offloading modalities

Responses to Question 3, an open-ended question that asked how offloading altered with the clinical course of the wound, showed both consistency and divergence in practices. Three key themes emerged: (i) overarching approaches to choosing offloading, (ii) drivers for altering offloading modality during wound management, and (iii) transitioning offloading modalities as a wound heals. Firstly, regarding approaches to choosing offloading, some participants reported selecting the maximal or most aggressive form of offloading therapy they thought a patient could tolerate and only altered this if there was a clear indication to do so. Other participants indicated that they choose more moderate offloading initially, which subsequently could be altered to a more aggressive option if there was a lack of progress with healing due to insufficient offloading. Secondly, and as expected, participants listed several drivers for altering offloading modality during wound management, including poor stability, compliance, tolerance, infection, difficulty with dressing changes, adverse effects to an offloading modality, gaining a patient’s trust and psycho-social factors, with a wide range of different types of offloading modalities being used as replacement. Thirdly, some participants reported that an offloading transition or step-down offloading was used, where a less intrusive offloading modality was applied, as wound healing progressed. Many participants acknowledged the final step of transitioning patients from ulcer offloading into maintenance offloading (e.g. shoes and orthoses) once full healing had occurred.

For Questions 4 to 7, clinicians were asked to rank how often practitioner-, patient-, intervention- and wound-related factors were taken into account in the selection of offloading modality using a 5-point Likert scale (never, not often, sometimes, often, always). Twenty-seven pre-specified factors were included, covering a broad range of issues. The median and the range for each factor are presented in Table 4. The data shows that 23 of the 27 pre-specified factors were either ‘always’ or ‘often’ considered and included issues such as evidence-based practice, restrictions on activities of daily living, features of the wound, expertise to apply the modality, patient activity levels, work needs and availability of medical backup. Three of the 27 factors that were ‘sometimes’ considered were bulkiness and/or weight of the intervention, application time and cost. Appearance was ‘not often’ considered.

**Table 4:** Frequency that pre-specified factors are taken into account in the selection of offloading

**Participant, patient, intervention and wound-related factors**	**Median**	**Range**
**Practitioner-related factors**		
Evidence-based practice	4	1 to 4
Personal experience/preference	3	2 to 4
Standard practice for your workplace*	3	0 to 4
Your expertise in prescribing/applying an intervention	4	1 to 4
The availability of staff to dedicate time required to apply/prescribe that intervention*	4	1 to 4
Whether an intervention will restrict wound care by the practitioner	4	1 to 4
**Patient-related factors**		
Whether an intervention will restrict activities of daily living (ADLs)	4	0 to 4
Whether an intervention will restrict hygiene	3	1 to 4
Whether an intervention will restrict wound care by the patient	3	1 to 4
How easy it is for the patient to use the intervention	4	2 to 4
Patient preference	3	0 to 4
How active the patient is	3	0 to 4
Patient work requirements	3	1 to 4
**Intervention-related factors**		
Cost	2	0 to 4
Whether an intervention may cause secondary complications	4	2 to 4
The availability of medical back up should secondary complications arise	4	0 to 4
Appearance of the intervention (i.e. looks)	1	0 to 2
Bulkiness and/or weight of the intervention	2	0 to 4
Whether an intervention will be well tolerated	3	2 to 4
How long an intervention will take to apply	2	0 to 4
Whether an intervention will cause gait instability	4	2 to 4
**Wound-related factors**		
Size of ulcer	4	2 to 4
Location of ulcer	4	0 to 4
Depth of ulcer	4	2 to 4
Associated foot deformity	4	2 to 4
Previous partial foot amputation	4	2 to 4
Number of ulcers	4	0 to 4

Notes: (i) n = 36 for all variables except n = 35*, (ii) scale used was from 0 – 4 (*Never = 0, Not often = 1, Sometimes = 2, Often = 3, Always = 4*).

### Barriers that exist to the use of non-removable casts or walkers

Participants were asked if they considered non-removable casts or walkers to be the gold standard for offloading this ulcer type (Question 8) and if applicable, the percentage of time that they used various types of non-removable modalities (Question 9). Thirty of 36 clinicians (83%) indicated that they did consider non-removable casts or walkers to be the gold standard for offloading non-infected, non-ischaemic plantar neuropathic foot ulcers. Three clinicians (8%) indicated that they did *not* consider this to be the case and the remaining 3 (8%) were unsure. Data reporting percentage of time various types of non-removable modalities were used (Question 9) was not analysed, as inconsistency of responses indicated that the question was not comprehended by all participants. As such, this data has not been reported.

For Question 10, participants were asked to report barriers they perceived existed to the use of non-removable offloading for this ulcer type. Responses reflected a host of inter-related issues that limit both the prescription and ongoing use of this type of offloading. Participants most commonly discussed patient-related barriers, followed by intervention-, wound- and practitioner-related barriers. The most common *patient-related barriers* were a lack of patient willingness to consent, which was partly due to difficulty in accepting that the device cannot be removed when wanted, plus issues around poor compliance (i.e. not following instructions, getting the offloading modality wet, irregular returns for cast changes, etc.). Of particular concern for patients living in hot and/or humid climates, and for patients where the device interfered with sleep, was that it could not be removed by patients. Negative impact on lifestyle (e.g. causing difficulty at work, restriction of self-care or choice of clothing, reduced mobility and walking), issues relating to accessing transport and driving, and patient ability to tolerate a non-removable device (including physical impairments, frailty, poor vision, obesity, etc.) were also of concern. Less reported but important barriers were religious and cultural issues (e.g. the need to be barefoot at home or wash feet for prayer), psychological health problems, intellectual capacity, and the suitability of the patient’s house and surrounds (e.g. stairs and hills).

*Intervention-related barriers* included; concerns over secondary iatrogenic complications such as new ulcers and falls, cost and application time. Less reported barriers were bulkiness and weight of the devices, access to materials and equipment and the limb length difference created with unilateral use. *Wound-related barriers* included reduced ability to access the wound for monitoring and dressing, managing high exudate, fluctuations in oedema and impact on skin condition. Although the question relating to wound-related barriers was framed around non-complicated neuropathic ulcers, it was acknowledged in the survey that a high-risk foot service would commonly manage more complex wounds in which non-removable offloading was often deemed unsuitable for clinical use. Situations where this might happen include ischemia, infection, unsuitable wound location and very large and/or deep wounds.

*Practitioner-related barriers* to using non-removable offloading related substantially to the availability of staffing resources. This was reported in relation to staff time and expertise required, flow-on pressure placed on a busy service, lack of on-call staff for patient emergencies and difficulties that could arise when patients required access to hospital emergency services. Barriers relating to clinician knowledge, skill, expertise and consistency in application were also reported, although these were not frequent.

Finally, Question 11 asked participants to estimate the percentage of time they were able to use their preferred offloading choice compared with offloading that was largely determined by other factors such as patient preference, cost, compliance, etc. Of the 35 participants that responded to this question, the mean (SD) response was that participants were able to use their preferred offloading 62% (±22%) of the time (range 13 to 100%).

## Discussion

The results of this survey provide a snapshot of offloading practices for plantar diabetes-related neuropathic foot ulcers in high-risk foot services around Australia. Participants indicated that they use a broad variety of offloading modalities in various frequencies when managing this type of ulcer, suggesting wide diversity in offloading practice. However, three offloading options were reportedly used the majority of the time by at least half or more of the participants surveyed. These modalities were padding (e.g. felt), followed by removable casts or walkers, then non-removable casts or walkers. The remaining 18 offloading options were used by fewer clinicians much less often.

The most outstanding finding from our survey, and one that is of key importance, was the relatively high proportion with which felt padding was used amongst the group sampled.

Felt padding ranked first in this survey for both the proportion of respondents that used the modality (94% for forefoot and 91% for hallux) and the percentage of time it was used (35% for both forefoot and hallux). This is of interest in light of the very limited evidence (e.g. from randomised trials) for its effectiveness for wound healing. A recent high quality systematic review [7] in this area found only two clinical trials using felt or felted foam padding that were eligible for inclusion and both trials were associated with a moderate to high risk of bias [16],[17]. Despite this, the results of this survey show that there is substantial support from clinicians working in high-risk foot services in Australia for the use of felt padding for the management of this type of ulcer, although the lack of high quality evidence highlights this as a priority area for future research.

The ‘first choice’ option recommended by clinical guidelines of total contact casts or other devices rendered irremovable ranked third in this survey for both the proportion of respondents that used the modality (68% for forefoot and 53% for hallux) and the percentage of time it was used (11% for both forefoot and hallux). Participants ranked removable casts or walkers slightly above this (73% for forefoot and 62% for hallux of participants, 16% of the time for both). This is not surprising considering the range of influences and barriers that participants reported in response to Questions 3 to 7 and Question 10 regarding selecting more aggressive offloading strategies.

It was also evident from participant responses to several survey questions that using offloading in ulcer healing is a complex and dynamic process where multiple variables are considered. That 23 of 27 (85%) pre-specified factors in Questions 4 to 7 were ‘often’ or ‘always’ considered indicates that the influence of an offloading modality on healing rate is only one factor that influences offloading selection. Given that current evidence-based guidelines are predominantly focused on the healing potential of an offloading modality, it is not surprising that a gap between evidence-based guidelines and clinical practice exists.

Responses to Question 3 further support the notion that there are multiple drivers for offloading selection. For example, context-specific factors were often reported by participants, including the need to be responsive to changing situations, scope for trial and error, and the requirement to customise offloading to suit the often unique needs of patients in this clinical population. Therefore, a diverse range of factors are considered, or serve as a barrier, when selecting offloading modalities, however these are poorly reflected in research and are not well articulated in clinical guidelines. As recommended by other authors, the findings of this survey suggest that research investigating a broad range of factors that might influence offloading success is required, including psycho-social, behavioural and health-related quality of life [7],[12].

The latter questions of the survey that related to using non-removable devices found that while 83% of participants indicated non-removable casts or walkers were the gold standard for offloading non-infected, non-ischaemic plantar neuropathic foot ulcers, numerous fundamental barriers to their use were reported. Of note were patient-related barriers related to gaining patient approval and compliance (which are likely to be linked to the reports of negative impact on lifestyle), issues around transportation/driving and patient ability to tolerate this type of device. The more common perceived barriers to using non-removable devices included the chance of causing iatrogenic complications such as new ulcers and falls, suitability for the wound type given foot ulcers are often complicated by ischaemia and/or infection, inability to monitor and dress the wound, and the impact on staff resources. Clinician expertise was not frequently reported as a barrier suggesting that either participants or the staff they worked with had the skills required to apply non-removable devices, such as total contact casts.

Several limitations of this study should be considered when interpreting the results. Firstly, there is the potential for bias including recall bias, survey bias and sampling bias. The researchers aimed to offset these biases by only surveying participants that used these modalities regularly (i.e. daily/weekly), as well as trialing and refining the survey using authentic testers. Secondly, given the complexity of some of the questions posed, there is the potential for misinterpretation and the possibility for error in some of the responses. Thirdly, it is recognised that clinicians working in the same service may undertake similar offloading practices, although we were interested in similarities (or differences) across services. Finally, the findings of this survey provide a snapshot of reported practice from the participants surveyed and caution should be exercised before broader generalisation is made to clinicians from other service types, patients from other settings, or countries.

## Conclusions

While there is evidence that non-removable casts or walkers are associated with superior healing for non-complicated neuropathic plantar foot ulcers, numerous barriers to their use were reported in this survey. Furthermore, a diverse range of factors (e.g. psycho-social) are considered when selecting offloading but these are poorly reflected in research and are not well embedded in clinical guidelines. Currently in the Australian high-risk foot setting, felt padding is reported as the most frequently used modality for offloading non-complicated plantar ulcers, however there is limited evidence for its use. Therefore, high quality randomised trials are needed to evaluate its effectiveness.

## Competing interests

AR is a member of the Editorial Board and KBL is Deputy Editor of the Journal of Foot and Ankle Research. It is journal policy that editors are removed from the peer review and editorial decision making processes for papers they have co-authored.

## Authors’ contributions

AR and KBL participated in the inception and design of the study, the data collection, statistical analysis and writing the manuscript. Both authors read and approved the final manuscript.

## Additional file

## Supplementary Material

Additional file 1: Offloading for neuropathic foot ulceration survey questions. Click here for file
