# Supplementary material for: A survey of offloading practices for diabetes-related plantar neuropathic foot ulcers
Source: J Foot Ankle Res. 2014 Aug 13;7:35. doi: 10.1186/s13047-014-0035-8 (PMC4332025; doi:10.1186/s13047-014-0035-8)
Supplement: Additional file 1: — Offloading for neuropathic foot ulceration survey questions. [file s13047-014-0035-8-S1.pdf]

Additional File 1:

Offloading for neuropathic foot ulceration

Survey questions

Questions 1 and 2 ask you to gauge the percentage of time that you use particular offloading modalities.

1. For the patients that you manage with <u>active neuropathic ulceration under the forefoot</u> (i.e. under the metatarsophalangeal joints), enter the percentage of time that you use the offloading modalities listed (please ensure that the total of percentages equals 100%).

If you use modalities in combination, indicate this in the 'other' option at the end of the table. In the space provided, list the offloading modalities that make up the combination/s you use, and the percentage of patients that you use each combination on. In this case, do not <u>also</u> enter the percentage usage under respective individual items in the table, we do not wish to count usage twice.

	% of patients used on
Bracing (e.g. ankle foot orthosis, patella tendon brace)	
Crutches	
Footwear – standard last and unmodified	
Footwear – off the shelf but extra-width and/or extra-depth and/or modified	
Footwear – fully custom-made	
Insoles/orthoses	
Non-removable cast or walker (e.g. total contact cast or walker made non-removable)	
Removable cast or walker	
Padding (felt, foam) with footwear (any type)	
Padding (felt, foam) with post-operative shoe	
Post-operative shoe (e.g. Darco)	
Offloading wound dressings	
Wheelchair	
Other - if yes indicate % and also provide details below, if not indicate 0%	

If you chose 'Other', indicate in the box below what modalities you use and the percentage of patients that you use them on. Please note that 'Other' can include single modalities or combinations of offloading modalities:

1.

2.

3.

4.

Add more if needed:

Examples:

1. Bed rest – used on 25% of patients

2. Combination of a non-removable walker and crutches – used on 10% of patients

2. For the patients that you manage with <u>active neuropathic ulceration under the hallux</u> (i.e. under the 1st interphalangeal joint), enter the percentage of time that you use the offloading modalities listed (please ensure that the total of percentages equals 100%).

If you use modalities in combination, enter them as an 'other' option at the end of the table. In the space provided, list the offloading modalities that make up the combination/s you use, and the percentage of patients that you use each combination on. In this case, do not <u>also</u> enter the percentage usage under respective individual items in the table, we do not wish to count usage twice.

	% of patients used on
Bracing (e.g. ankle foot orthosis, patella tendon brace)	
Crutches	
Footwear – standard last and unmodified	
Footwear – prefabricated but extra width and/or depth and/or modified	
Footwear – fully custom made	
Insoles/orthoses	
Non-removable cast or walker (e.g. total contact cast)	
Removable cast or walker	
Padding (felt, foam) with footwear (any type)	
Padding (felt, foam) with post-operative shoe	
Post-operative shoe (e.g. Darco)	
Offloading wound dressings	
Wheelchair	
Other - if yes indicate % and also provide details below , if no indicate 0%	

If you chose 'Other', indicate in the box below what modalities you use and the percentage of patients that you use them on. Please note that 'Other' can include single modalities or combinations of offloading modalities:

1.

2.

3.

4.

Add more if needed:

Examples:

1. Bed rest – used on 25% of patients

2. Combination of a non-removable walker and crutches – used on 10% of patients

3. We understand that choice of offloading may change as a wound alters or heals. If this is a valid comment about your practice, summarise in the box below the most common changes in offloading selection that you experience in the clinical course of a wound.

Questions 4 to 7 ask you to rank how practitioner-, patient-, intervention- and wound-related factors influence your selection of an offloading modality for the management of active forefoot or sub-hallux neuropathic ulcers.

4. How often do you take into account the following *practitioner-related factors* in the selection of an offloading modality, when treating active forefoot or sub-hallux neuropathic ulcers? CLICK ON RELEVANT BOXES TO CHECK.

	Never	Not often	Sometimes	Often	Always
Evidence-based practice	☐	☐	☐	☐	☐
Personal experience/preference	☐	☐	☐	☐	☐
Standard practice for your workplace	☐	☐	☐	☐	☐
Your expertise in prescribing/applying an intervention	☐	☐	☐	☐	☐
The availability of staff to dedicate time required to apply/prescribe that intervention	☐	☐	☐	☐	☐
Whether an intervention will restrict wound care by the practitioner	☐	☐	☐	☐	☐

5. How often do you take into account the following *patient-related factors* in the selection of an offloading modality when treating active forefoot or sub-hallux neuropathic ulcers? CLICK ON RELEVANT BOXES TO CHECK.

	Never	Not often	Sometimes	Often	Always
Whether an intervention will restrict activities of daily living (ADLs)	☐	☐	☐	☐	☐
Whether an intervention will restrict hygiene	☐	☐	☐	☐	☐
Whether an intervention will restrict wound care by the patient	☐	☐	☐	☐	☐
How easy it is for the patient to use the intervention	☐	☐	☐	☐	☐
Patient preference	☐	☐	☐	☐	☐
How active the patient is	☐	☐	☐	☐	☐
Patient work requirements	☐	☐	☐	☐	☐

6. How often do you take into account the following *intervention-related factors* in the selection of offloading modality when treating active forefoot or sub-hallux neuropathic ulcers? CLICK ON RELEVANT BOXES TO CHECK.

	Never	Not often	Sometimes	Often	Always
Cost	☐	☐	☐	☐	☐
Whether an intervention may cause secondary complications	☐	☐	☐	☐	☐
The availability of medical back up should secondary complications arise	☐	☐	☐	☐	☐
Appearance of the intervention (i.e. looks)	☐	☐	☐	☐	☐
Bulkiness and/or weight of the intervention	☐	☐	☐	☐	☐
Whether an intervention will be well tolerated	☐	☐	☐	☐	☐
How long an intervention will take to apply	☐	☐	☐	☐	☐
Whether an intervention will cause gait instability	☐	☐	☐	☐	☐

7. How often do you take into account the following *wound-related factors* in the selection of offloading modality when treating active forefoot or sub-hallux neuropathic ulcers? CLICK ON RELEVANT BOXES TO CHECK.

	Never	Not often	Sometimes	Often	Always
Cost	☐	☐	☐	☐	☐
Size of ulcer	☐	☐	☐	☐	☐
Location of ulcer	☐	☐	☐	☐	☐
Depth of ulcer	☐	☐	☐	☐	☐
Associated foot deformity	☐	☐	☐	☐	☐
Previous partial foot amputation	☐	☐	☐	☐	☐
Number of ulcers	☐	☐	☐	☐	☐

8. Do you consider non-removable offloading, such as total contact casts (TCCs), to be the gold standard in offloading non-infected, non-ischaemic, plantar neuropathic foot ulcers? CLICK ON RELEVANT BOX TO CHECK.

Yes ☐ No ☐ Unsure ☐

In the next question (question 9) we would like to know more about the types of non-removable casts or walkers you use, if applicable.

If you do not use non-removable casts or walkers for offloading ulcers, please go to question 10.

9. If you use non-removable casts or walkers for offloading active plantar neuropathic ulceration, indicate the percentage of time that you use the devices listed (please ensure that the total of percentages equals 100%).

	%
Total contact cast	
Non-removable walker (e.g. prefabricated walker made irremovable)	
Other - if yes indicate % and also provide details below, if no please indicate 0%	

If you chose 'Other', please indicate in the box below what modalities you use and the percentage of patients that you use them on.

1.

2.

3.

4.

Add as needed:

10. What barriers do you believe exist, if any, to the use of non-removable offloading (i.e. total contact casts or walkers made non-removable) in the management of non-infected, non-ischaemic, plantar neuropathic foot ulcers? ENTER YOUR ANSWER IN THE BOX BELOW.

11. In practice, what percentage of time do you estimate that you are able to use your preferred offloading choice as compared to offloading selected due to other factors (such as patient preference, cost, compliance, etc.)?

ENTER THE % IN THE BOX BELOW.

You have now reached the end of this survey.

Thank you very much for your input.
